# Large-scale analysis of human heavy chain V(D)J recombination patterns

**DOI:** 10.1186/1745-7580-4-3

**Published:** 2008-02-27

**Authors:** Joseph M Volpe, Thomas B Kepler

**Affiliations:** 1 Center for Computational Immunology, Duke University, Durham, NC, USA; 2 Department of Immunology, Duke Univeristy Medical Center, Durham, NC, USA; 3 Department of Biostatistics and Bioinformatics, Duke Univeristy Medical Center, Durham, NC, USA; 4 Institute of Statistics and Decision Sciences, Duke Univeristy, Durham, NC, USA; 5 Computational Biology and Bioinformatics Graduate Program, Institute for Genome Sciences and Policy, Duke Univeristy, Durham, NC, USA

## Abstract

**Background:**

The processes involved in the somatic assembly of antigen receptor genes are unique to the immune system and are driven largely by random events. Subtle biases, however, may exist and provide clues to the molecular mechanisms involved in their assembly and selection. Large-scale efforts to provide baseline data about the genetic characteristics of immunoglobulin (Ig) genes and the mechanisms involved in their assembly have recently become possible due to the rapid growth of genetic databases.

**Results:**

We gathered and analyzed nearly 6,500 productive human Ig heavy chain genes and compared them with 325 non-productive Ig genes that were originally rearranged out of frame and therefore incapable of being biased by selection. We found evidence for differences in n-nucleotide tract length distributions which have interesting interpretations for the mechanisms involved in n-nucleotide polymerization. Additionally, we found striking statistical evidence for pairing preferences among D and J segments. We present a statistical model to support our hypothesis that these pairing biases are due to multiple sequential D-to-J rearrangements.

**Conclusion:**

We present here the most precise estimates of gene segment usage frequencies currently available along with analyses regarding n-nucleotide distributions and D-J segment pair preferences. Additionally, we provide the first statistical evidence that sequential D-J recombinations occur at the human heavy chain locus during B-cell ontogeny with an approximate frequency of 20%.

## Background

Immunoglobulins (Ig) are the primary humoral effector molecules of the adaptive immune system of jawed vertebrates. An Ig molecule is a homodimer of heterodimers where each heterodimer is made from one heavy chain and one light chain protein. The genes for both chains are encoded by ligated gene segments genetically rearranged during a process known as V(D)J recombination [1,2]. In humans, there are approximately 50 known functional V (variable) segments [3-6], 27 known functional D (diversity) segments [3,7,8], and six known functional J (joining) segments [3,8,9] available within a single locus for assembly into heavy chain genes. The locus is located near the long-arm telomere of chromosome 14 and extends inward toward the centromere with the V segments at the 5' end followed by the D segments and then J segments.

During recombination, non-templated (n)-nucleotides may be added between adjoining gene segments by terminal deoxynucleotidyl tranferase (TdT) [10]. These nucleotides become part of complementarity determining region 3 (CDR3), a section of the gene that encodes one of the primary antigen binding loops in the resulting protein. This loop is responsible for much of the population diversity of Ig molecules since it spans the 3' end of the V segment through to the 5' end of the J segment, entirely encompassing the rearranged D segment. Together, the mechanisms that control n-nucleotide addition and the rearrangement of various gene segment combinations enable the generation of over 10^7 ^different protein specificities. The processes that produce the Ig repertoire are largely random, but the biases (deviations from strict randomness) that do exist potentially provide clues about the mechanisms by which these processes operate. Several studies have been published reporting analyses of these biases. Using 71 productive Ig rearrangements from a single individual, Brezinschek et. al. [11] characterized V, D, and J segment usage by PCR analysis of genes from unstimulated B-cells, providing the first evidence for biased gene segment usage within an individual's immature B-cell repertoire. They showed, in particular, that the VH3 family is differentially over-represented among VH gene segments, and that JH6 is expressed more frequently than any of the other segments. In a follow-up study [12] the investigators used samples from two human subjects to study both productive and non-productive Ig rearrangements. By including non-productive sequences and comparing these unselected rearrangements to productive rearrangements subject to selection, they were able to attribute the differential usage to selection. Specifically, they showed that a certain VH4 family segments appeared to be selectively suppressed.

A 2001 study by Rosner et. al. [13] used cells from ten human subjects to study CDR3 length differences between mutated and non-mutated Ig genes. Their analysis led them to hypothesize that B-cells bearing Ig with shorter CDR3 are selected for antigen binding. In the course of this study, the authors established statistical baselines for typical n-nucleotide tract lengths in the V-D and D-J junctions of Ig genes and provided some of the first statistics regarding D gene segment usage frequency and CDR3 length in the adult human Ig repertoire. More recently, Souto-Carneiro et. al. [14] gathered Ig sequences from several studies, including the aforementioned Brezinschek study, to characterize CDR3 structure statistically using more sequences than had been previously available in a single study. They developed specialized software for the analysis of CDR3 D segment usage, D segment reading frame, and amino acid composition and provide one of the most complete statistical analyses of D gene segment usage to date, including evidence for the use of the controversial "irregular D segments" [8].

Our approach involves using a much larger set of Ig genes than was previously possible. Large scale initiatives to study Ig repertoire biases have only recently become tractable. Developments in laboratory methods and sequencing technologies have facilitated rapid production of large genetic datasets for Ig. The parallel rise of bioinformatics and systems biology has promoted methods for storage, analysis, and sharing of those data. Genbank, for example, currently holds over 20,000 human Ig records. We are fortunate to have access to this profusion of Ig sequence data as it presents an opportunity to study statistically the genetic and molecular details of Ig using a large dataset that only recently has become manageable. We present here the results of a comprehensive characterization of nearly 6,500 human Ig genes in terms of V, D, and J gene segment usage, n-nucleotide addition, and CDR3 length, and an analysis of the molecular mechanisms involved in Ig gene creation. We include a detailed characterization and comparison of those sequences to 325 non-functional rearrangements. One of our more striking findings is the existence of strong pairing preferences among D and J gene segments. We hypothesize that these results may be due to repeated sequential rearrangement of D and J segments and present a statistical model that illustrates the efficacy of this mechanism for producing the observations. In addition, we have found that the n-nucleotide tract lengths in both he V-D and D-J junctions are well-fit by a negative binomial distribution. Differences in tract length distributions between these two junctions are characterized by specific differences in the parameters of the distribution, which can be interpreted in terms of mechanisms of n-nucleotide polymerization.

## Results

### Preferred pairing among gene segments

We performed contingency table analyses to investigate whether there is preferred gene segment pairing between D and J segments in the P genes. We tabulated the frequency of occurrence of each D-J pair and used a contingency table to compare these frequencies with those expected under the null hypothesis of independent selection. The extremely low p-value (*p *< 10^-50^) for the chi-square analysis indicates that the D and J segments are not independent. To measure the degree of departure from independence of each pair, we calculated adjusted residuals, which are approximately independent and distributed as standard normals [15]. So, values greater than 1.96 or less than -1.96 for particular D-J pairs represent a significant departure from the expected value at a 95% confidence level and are therefore evidence for a correlation between that particular D and J segment. We analyzed the P sequences only since the number of NP sequences was insufficient for this analysis.

**Figure 1:**
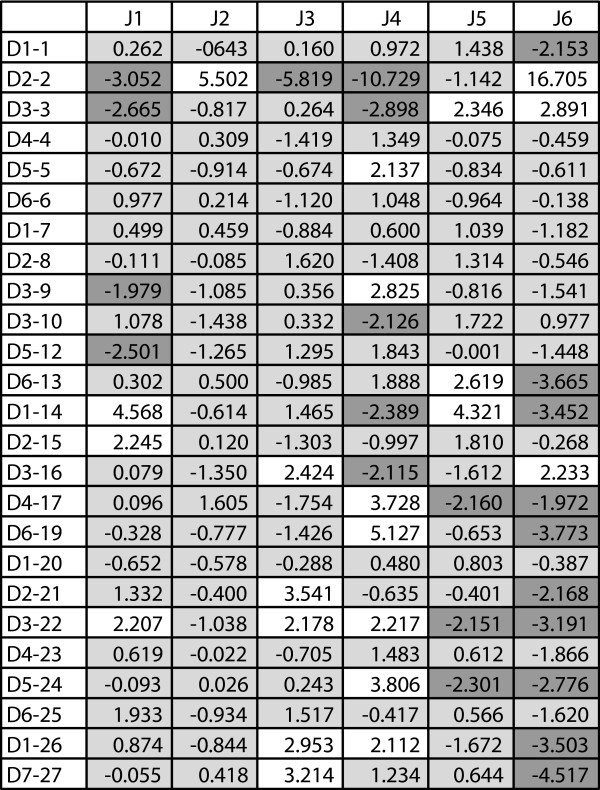
**Adjusted Residuals for D-J Segment Pairings**. A heat map showing adjusted residual values for D-J segment pairings based on contingency table analysis of the P sequence data. Adjusted residuals are approximately independent and distributed as standard normals. Values greater than 1.96 (white) or less than -1.96 (dark gray) represent a significant departure from the expected value at a 95% confidence level.

Our data show that certain pairs of D-J segments have frequencies significantly different from what is expected under the null hypothesis (Table 1, Fig. 1). For example, based on the marginal frequencies of D2-2 and J6 (13.3% and 25.3%, respectively), we expected a frequency of 3.3% for the D2-2/J6 pair. The observed frequency, however, was 6.5%, an increase of 94% over what was expected. Our segment pair observations highlight an interesting pattern of D-J correlations within the data. Several 5' D segments showed increased frequency of pairing with the most 3' J segments (J5 and J6) and decreased frequency of pairing with closer (chromosomal distance) J segments (J1-J4). Some 3' D segments, however, showed increased frequency of pairing with the closest J segments (J1-J4), but a decreased frequency for the furthest J segments, J5 and J6. These findings led us to the hypothesize that multiple successive D-J recombinations may occur prior to adjoining a V segment to the D-J pair. This hypothesis has been put forth before, but little evidence has been offered for this occurence in humans [14,16,17].

**Table 1:** D Segment Frequencies. Observed counts and relative frequencies of individual D segment usage in the P and NP datasets.

	P sequences		NP sequences	
	Obs.	Rel. freq.	Obs.	Rel. freq.

D1-1	133	0.020	2	0.006
D2-2	811	0.125	76	0.234
D3-3	498	0.077	26	0.080
D4-4	78	0.012	1	0.003
D5-5	192	0.030	8	0.025
D6-6	141	0.022	5	0.015
D1-7	99	0.015	3	0.009
D2-8	129	0.020	4	0.012
D3-9	246	0.038	6	0.018
D3-10	547	0.084	17	0.052
D5-12	144	0.022	3	0.009
D6-13	295	0.045	22	0.068
D1-14	56	0.009	3	0.009
D2-15	268	0.041	22	0.068
D3-16	313	0.048	17	0.052
D4-17	263	0.041	10	0.031
D6-19	425	0.065	14	0.043
D1-20	10	0.002	0	0.000
D2-21	184	0.028	8	0.025
D3-22	527	0.081	28	0.086
D4-23	94	0.014	4	0.012
D5-24	153	0.024	3	0.009
D6-25	26	0.004	0	0.000
D1-26	364	0.056	13	0.040
D7-27	101	0.016	7	0.022
D0-IR	72	0.011	5	0.015
D1-IR1	94	0.014	6	0.018
D1-OR15	11	0.002	1	0.003
D2-IR2	33	0.005	2	0.006
D2-OF15	88	0.014	6	0.018
D3-OR15	47	0.007	2	0.006
D4-OR15	30	0.005	0	0.000
D5-OR15	18	0.003	1	0.003
	6490		325	

**Figure 2:**
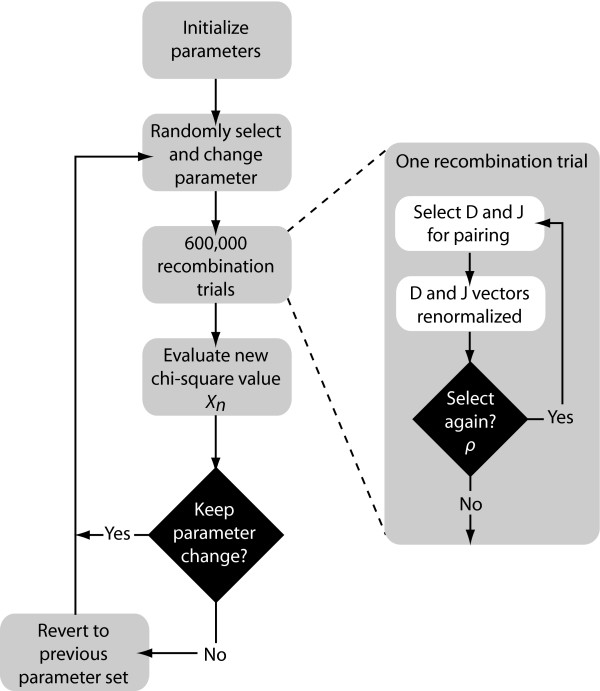
**Estimation Algorithm Depiction**. Flow diagram depicting the steps for estimating the multiple recombination parameters in our statistical model. Rho (*ρ*) is the parameter for multiple recombination; it represents the probability of a subsequent recombination occurring given that one just occurred and that segments are available for another recombination. Changes to the parameters are accepted stochastically according to the Metropolis-Hastings criterion: with probability 1 if the new chi-square ($\chi_{new}^{2}$) value is lower than the old value ($\chi_{old}^{2}$), or with probability exp(0.5($\chi_{old}^{2}-\chi_{new}^{2}$)) (37).

To test this hypothesis, we developed a statistical model to estimate the frequency of multiple sequential recombinations. The parameters of this model are the relative probabilities for choosing a given segment and a parameter that gives the probability for making a subsequent recombination at every stage (see methods). We ran the algorithm for 300,000 iterations in which each iteration included 600,000 recombination trials were performed (Fig. 2). We found the best fit of our data to the model at *ρ *= 0.198. At this multiple recombination rate, the model produced a chi-square value of 503. This is very statistically different from 635 (*p *< 10^-30^; chi-square test with 1 degree of freedom), the chi-square value observed when *ρ *= 0.

### CDR3 statistics

Our data show statistically significant differences in the length of CDR3 between the P and NP sequences (*p *< 10^-10^). The P sequences have a shorter mean CDR3 length of 15.49 amino acids while the NP sequences have a mean length of 18.00 amino acids. In the V-D junction, an average of 7.86 and 9.78 n-nucleotides were added to the P and NP sequences, respectively: a statistically significant difference (*p *< 0.001). For the D-J junction, the data show statistically different averages of 7.04 and 8.26 n-nucleotides for the P and NP sequences, respectively (*p *< 0.01).

**Figure 3:**
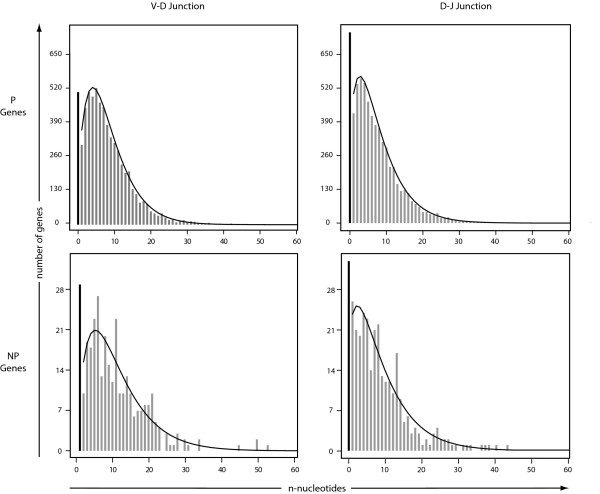
**Fitting Plots of n-Nucleotide Data**. Plots of the observed n-nucleotide data for both the P and NP genes in both the VD and DJ junctions fit to a zero-inflated negative binomial distribution.

**Figure 4:**
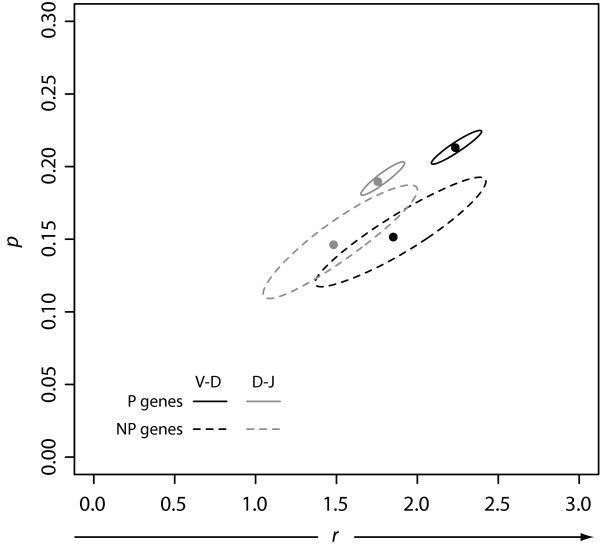
**Confidence Regions for n-Nucleotide Data Fits**. We fit our observed n-nucleotide addition data to the negative binomial distribution, and calculated both the maximum likelihood estimators plotted at (r, p) and the corresponding confidence regions.

Plots of the observed n-nucleotide frequencies resembled plots of a zero-inflated negative binomial distribution. The negative binomial distribution is a discrete probability distribution for the number of independent Bernoulli trials required to achieve a fixed number, *r*, of successes. For both P and NP data of n-nucleotide additions in both the V-D and D-J junctions, we fit our data to the negative binomial distribution and calculated the maximum likelihood estimator (MLE) for the parameters *r *and *p*, where *p *is the probability of getting a success in any given trial. (Table 2, Fig 3). We then calculated 95% confidence regions (Fig 4). We found that for the P sequences, *r *< 2 for the D-J junction but *r *> 2 for the V-D junction.

**Table 2:** Negative Binomial Parameters. Negative binomial parameters *r *and *p *produced from fits of the observed n-nucleotide data from the V-D and D-J junctions for both the P and NP gene sets. In our model, we interpret *r *to mean the number of detachments TdT experiences from the DNA.

	Junction	*r*	*p*	Mean n addition
P	V-D	2.24	0.21	7.86
	D-J	1.76	0.19	7.04
NP	V-D	1.85	0.15	9.78
	D-J	1.48	0.15	8.26

### Gene segment usage frequencies

**Figure 5:**
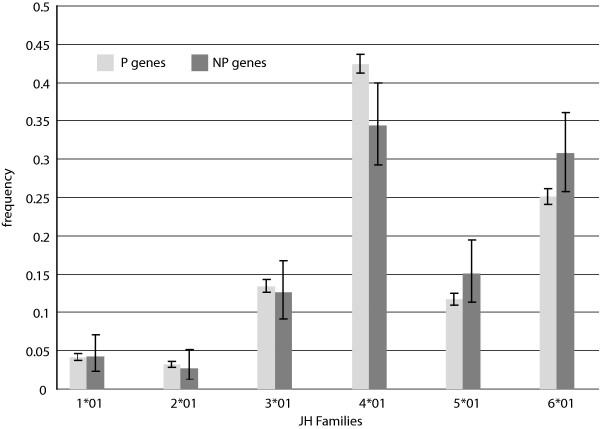
**J Gene Segment Usage Frequencies**. Observed relative frequencies of JH gene segment usage in the P and NP gene sets.

The ability to detect biases statistically is made easier when the number of total categories is small. The J locus has fewer gene segments than either of the other heavy chain loci, and thus provides the best opportunity for the discovery of such bias in gene segment usage. Indeed, we find very strong departure from uniform segment usage in both P and NP sets (*p *< 10^-12^) which both show a strong preference for J4 and J6 and substantially reduced frequency of J1 and J2 (Fig. 5). There are also differences in relative frequencies of J segment usage between P and NP genes (*p *= 0.03), with J4 under-represented by 18% among NP genes relative to P, and J5 and J6, over-represented by 27% and 21% in NP compared to P, respectively.

**Figure 6:**
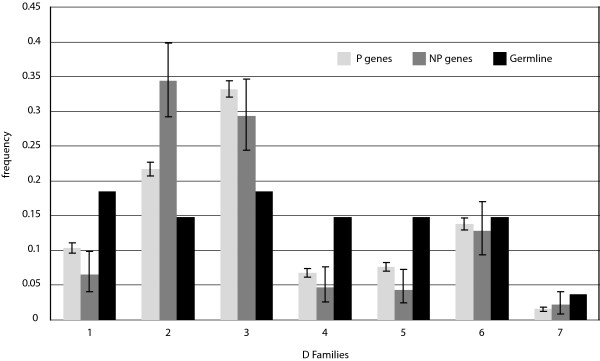
**D Gene Segment Usage Frequencies**. Relative observed frequencies of DH gene segment usage by family in the P and NP gene sets, and comparison to germline complexity of each gene segment family. The germline complexity refers to the number of segments within the locus assigned to each family.

D segments, which outnumber J segments by more than a factor of four, are organized into seven families based on sequence homology. At a family level, we compared usage of segments of both the P and NP sets to the genomic complexity of each family, which is the number of segments assigned to each family within the locus, and found a significant departure from these proportions as well (*p *< 10^-12^) (Fig. 6). Again, we observe statistically significant differences in relative frequencies of usage between the P and NP sequences at both the family and individual gene segment levels (*p *< 10^-5^). Family D2 is over represented among NP genes by 55% relative to the P sequences, but families D4 and D5 are under represented by 29% and 41%, respectively, relative to the P sequences (Fig. 6). Individually, we again find a strong departure from uniform segment usage in both the P and NP sets (*p *< 10^-12^). The most notable disparity is with segment D2-2, which is significantly over represented in the NP genes by 82% relative to the P genes. There was not a statistically significant difference in the number of inverted D segments observed between the P and NP genes.

**Figure 7:**
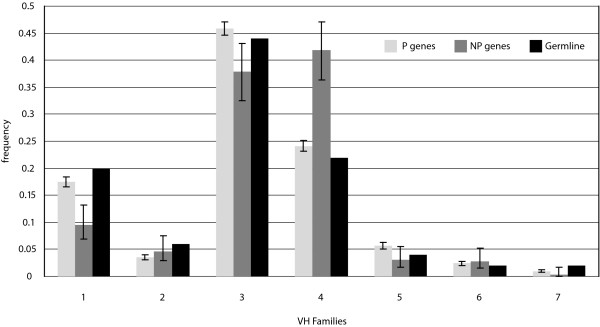
**V Gene Segment Usage Frequencies**. Observed relative frequencies of VH gene segment usage by family in P and NP sequences, and comparison to germline complexity of each gene segment family.

Like the D segments, V segments are also classified into seven families based on sequence homology. The frequencies of V gene segment usage by family in the P and NP gene sets, compared to the genomic complexity of the V locus, are shown in figure 7. Though the observed frequencies produce a very significant rejection of the hypothesis that the usage of V segments in the P and NP gene sets are exactly proportional to the number of genes in each family (*p *< 10^-10^), the P genes more closely resemble the genomic complexity of the V locus than do the NP genes. Relative to the P genes, the NP genes differ significantly (*p *< 10^-10^), with usage frequencies 44% and 17% below what is expected for families V1 and V3, respectively, but 67% greater than what is expected for family V4. Concerning individual segments, both P and NP genes used segments V3-23 and V4-34 most frequently, though V3-23 was the top segment in the P genes, but second to V4-34 in the NP genes.

## Discussion

We provide here the most precise estimates of gene segment usage frequency currently available. The quantity of data that we assembled and analyzed has enabled us to estimate V, D, and J segment usage frequencies with tight confidence intervals. These data potentially give insight into the structural basis for differential segment usage in terms of either raw expression or somatic selection, though such elucidations are left for further research.

In addition, comparison of our results with previously published usage frequencies provides validation of our data collection methods and confidence that our P sequence dataset is representative of natural diversity as intended. In particular, an extensive study of Ig CDR3 diversity based on de novo sampling of Ig using a primer for a single VH gene shows D segment usage results remarkably similar to our own, based on a Spearman rank correlation score of 0.93 [18]. This in spite of the fact that D segments are notoriously challenging to identify within Ig genes due to recombination site choice, flanking 5' and 3' n-nucleotide addition [10], and somatic mutation [19-21]. With J segments, furthermore, our data are consistent with published findings that indicate that segment J4 is used most frequently, followed in descending order by segments J6, J5, J3, J1, and J2 [11,22,23] (Fig. 5).

For V segments, our data again provide statistical evidence in support of published findings. With individual segments, our data support previous results showing that segment V3-23 is the most frequently used [11] in productive rearrangements, and that gene V4-34, which we found to be used second most frequently, has high usage within adult peripheral lymphocytes [23]. Like the J segments, individual segment usage can vary, but in spite of that, segment usage at the family level approximates expected usage based on literature. Our data support findings that show that segments in family V3 are used most frequently, followed in descending order by V4, V1, V5, V2, V6, and lastly V7 [11,12]. We have also shown consistency with findings that, with some variation, the distribution of V gene usage by family shows similarity to germline complexity of the known segments [11] (Fig. 7). Our data showed this to be especially true for families V1, V3, and V4.

The NP sequences showed an enhancement of segment usage from family V4 at the expense of segments from family V1, due primarily to a 67% increase in usage of segment V4-34 from what was expected. Segment V4-34 has been reported to be over-represented in the adult human repertoire [24], and has also been implicated in generating autoreactive B-cells in SLE patients and against cold agglutinins [25-27]. Since the NP sequences are not subject to selection, those sequences coding for autoreactive receptors would not be deleted from the repertoire. Also, V4-34 has been shown previously to be limited by selection in the expressed human Ig repertoire due to lowered usage of this segment between IgM and IgG populations [28]. Thus, V4-34 is likely not enhanced in autoimmune disorders, but instead is selectively limited in the P sequences.

Having validated our data collection methods, we focused on analyzing the genetic mechanisms involved in V(D)J recombination. One such mechanism is n-nucleotide addition by TdT. The zero-inflated negative binomial model fits these data well enough for us to seek an interpretation of its three parameters. We develop this interpretation in terms of two states: TdT attached to one of the unjoined DNA ends, or unattached. The probability that TdT never attaches is the first parameter, the zero-inflation factor. When attached, TdT either adds another nucleotide or becomes detached, with probabilities *p *and 1 - *p*, respectively. In this context, the final parameter, *r*, has a natural interpretation as the number of times TdT detaches before the joint is closed.

We found that for the P sequences, *r *< 2 for the D-J junction but *r *> 2 for the V-D junction (Table 2). This pattern is consistent with a greater TdT concentration during the V-D joining process relative to that during the D-J process.

Studies of TdT expression during B-cell ontogeny show high levels TdT mRNA during the pro-B and late pro-B stages of development – the stages in which the D-to-J and the V-to-DJ rearrangements occur, respectively [29,30]. Specifically, it has been shown that TdT expression is upregulated as the B-cell moves from the pre-pro-B stage, undergoing D-to-J recombination, and that expression peaks as the V-to-DJ rearrangement occurs in the late pro-B stage [30]. TdT expression then quickly declines as the cell progresses into the pre-B stage. This observation is consistent with our result, that there are more detachments (and hence more attachments) before end-joining in the V-D junction relative to the D-J junction (Table 2).

We also investigated the mechanisms involved in gene segment recombination. Our findings regarding D-J segment correlations raise an interesting hypothesis that multiple successive D-J rearrangements may occur prior to recombination with a V segment. Previously, Reth et.al. tested the possibility of this hypothesis in murine 300-19 cells cultured in vitro by assaying for the presence of a designated D-J insert and found that such multiple successive recombinations can and do occur [16]. Other studies analyzing nonproductive human Ig rearrangements have hypothesized, based on their observations, that multiple successive D-J rearrangements at the human heavy locus are likely [14,17]. We here provide evidence for this hypothesis for human Ig. This rearrangement mechanism differs from that observed in receptor editing in the heavy chain via *V*_*H *_replacement [31] or at the light chain loci by secondary de novo rearrangements [32,33]. Our analysis suggests that multiple D-J rearrangements may occur up to 15% of the time prior to the V-to-DJ rearrangement, with each successive D-J recombination replacing the previous one via excision.

The processes involved in D-J recombination are complex and likely require more parameters to better model the system. Still, the results of our modeling, with such extreme differences in p-value and chi-square values, are sufficient to support our hypothesis for the observed patterns in our P sequences. These data provide the first statistically supported observations of multiple successive recombinations in productive human Ig sequences. Considering V-D pairings, we did not perform a similar contingency table analysis since the greater number of possible pairs dramaticallyreduces the statistical power. For the NP sequences, the relatively low number of sequences in this set did not allow for this analysis.

These analyses prompted us to speculate about the observed J segment frequencies. Our multiple recombination model can help explain the lower usage frequency of segments J1 and J2, but prompts one to question why V5 is not used as frequently as V6, yet instead has a similar frequency to V3. Of the remaining four segments, J4 and J6 are used most frequently, followed by J5 and J3. It is possible that there are structural reasons for these observations concerning DNA access and histone acetylation. We propose, however, that the observed trends may instead be due to selection for tyrosine residues. Analyses of the 5' portion of the functional J segments, up to the invariant tryptophan residue, show that both J3 and J5 lack tyrosine residues, while J4 has two and J6 has five. Tyrosine has biochemical and structural properties that make it beneficial in protein binding interfaces, such as CDR [34]. Also, studies of amino acid profiles in human Ig have shown that tyrosine is one of the most abundant residues found in CDR, and specifically within CDR3, it locates most often at the C-terminus end of the CDR3 loop [34,35]. Any residues contributed to CDR3 by J segments would be found at the C-terminus end of CDR3. The desirability of tyrosine residues and their frequent location at the 3' end of CDR3 suggests biased selection toward proteins comprised of J segments that contribute such residues, namely J4 and J6.

With regard to CDR3 length, we found that the P sequences had a statistically shorted mean compared with the NP sequences. The higher mean CDR3 observed in the NP sequences may be due to a lack of selection. It has been previously shown that negative selection occurs in the bone marrow against B-cells presenting Ig with long CDR3 [36]. This may be because Ig with long CDR3 have been correlated with polyreactive specificity, including specificity for self peptide [37]. Since the NP sequences are not subject to selection in the bone marrow, these data provide evidence that negative selection restricts CDR3 length in the human Ig repertoire.

## Conclusion

We applied a statistical approach to the study of the mechanisms involved in Ig gene formation by utilizing the wealth of publicly available data. Amassing sequence data from Genbank may be precarious. Yet, our observations of gene segment frequencies aligned well with previous reports, validating our approach and allowing us to provide novel statistical evidence for interesting mechanisms that shape the human Ig heavy chain repertoire.

We provide here the most precise estimates of human heavy chain gene usage frequency currently available. Additionally, we provide here the first statistical evidence in humans for sequential D to J recombination at the human heavy chain locus.

## Methods

### DNA sequences

We set out to compile a of human immunoglobulin heavy chain gene sequences that is representative of natural immunoglobulin diversity, excluding clonally related genes and genes of autoimmune and perinatal origin. To do so, we submitted the search "human [orgn] heavy [titl] immunoglobulin [titl]" to the Genbank nucleotide database which returned 16,870 results, downloaded the DNA sequences, preprocessed and filtered them as described below, and analyzed them for gene segment usage, point mutations, n-nucleotide addition, and recombination junctional diversity. The automated analysis was performed using our in-house software SoDA [38].

### Filtering

We filtered the dataset to remove clonal duplicates, which we defined to be those sequences that were inferred to use the same V, D, and J gene segments, had the same inferred CDR3 length, and have nearly sequential Genbank accession numbers. Where groups of clonally related genes were identified, a single representative was chosen at random and the others were omitted. We also filtered out sequences that, by their own Genbank annotations, indicated origin from neonates or cordblood because of known gene segment and CDR3 biases [36,39-41]. The dataset was also filtered to remove any sequences that may be autoreactive as indicated by the presence one of any of the following words in the Genbank record: "self-reactive", "anti-self", "lupus", "rheumatoid", "sjogren", "diabetes", "sclerosis", "wegener", "crohn", "addison", "scleroderma", "grave", "psoriasis", "celiac", "vasculitis", "colitis", and "thyroiditis". We then grouped the sequences by study of origin according to accession number and removed large sets of sequences derived from the same study to prevent the biases of any single study from having a disproportionate impact on the study as a whole.

### Classification by Productivity

The dataset was then divided into three groups on the basis of their inferred original, pre-somatic mutation productivity. We classified those sequences that had no stop codons and both invariant V cysteines and the invariant J tryptophan in-frame and intact as productive (P). Those that appeared to have been originally rearranged out of frame by virtue of the V segment being out of frame with the J segment, excluding indels, were classified as non-productive (NP). All others were classified as indeterminate and omitted from further consideration. The final set of productive genes contained 6490 sequences; the final set of non-productive genes contained 325 sequences.

### D-J Recombination Statistical Model and Algorithm

We developed a statistical model and estimated its parameters using a Markov Chain Monte Carlo (MCMC) method to study observed D and J segment pairing preferences. We fit our model to the observed data by estimating probability vectors for D and J segment usage and a multiple recombination rate (MRR) parameter, *ρ*. Each component of the probability vectors gives the relative probability that the corresponding segment will be chosen during the recombination process at any stage. The MRR is the probability of a subsequent recombination occurring given that one just occurred and that segments of the same type remain to produce another recombination. The D and J parameter vectors are initialized to the marginal frequencies calculated from the observed D-J pair frequencies, and *ρ *is initialized to 0.10. The algorithm begins by first running a set of 600,000 recombination trials using the initialized D and J vectors. When the trials are complete, the D-J pair frequencies are compared with the observed frequencies and a chi-square value is established. One of the parameters in the D or J vectors, or *ρ*, is then selected at random and altered slightly and a new set of 600,000 primary recombination trials begins. For each primary recombination, a D and J segment are initially selected. All intervening segments between those selected are designated as unavailable and the probabilities of the remaining segments are recalculated, normalizing them to represent the new restricted set of available segments. Then, with probability *ρ*, a subsequent recombination may occur. If this secondary recombination does occur, the probabilities of the remaining segments are again normalized. Subsequent recombinations may continue to occur in this manner provided that there are segments available to recombine. If at any stage, the algorithm does not choose to make a subsequent rearrangement, the process terminates. It also terminates when no more segments can be recombined. At the completion of all 600,000 trials, the D-J pair frequencies of the trials are compared with the observed values, and a chi-square value is computed. The new parameter values are accepted stochastically according to the Metropolis-Hastings criterion: with probability 1 if the new chi-square ($\chi_{new}^{2}$) value is lower than the old value ($\chi_{old}^{2}$), or with probability exp(0.5($\chi_{old}^{2}-\chi_{new}^{2}$)) [42].

Otherwise, the algorithm reverts back to the previous set of parameters. This enables the algorithm to occasionally accept non-improving moves and thereby avoid being trapped in local minima. The algorithm then repeats, altering another parameter and performing a new set of trials (Fig. 5). The output of the algorithm represents a sample from the Bayesian posterior density on the parameters.

## Authors' contributions

JMV gathered and analyzed the data, computationally implemented the statistical model, and drafted the manuscript. TBK determined, and where necessary, developed the appropriate statistical strategies needed for the presented analyses. Both authors contributed to the final writing of the manuscript.
